# Comparison of Two-Dimensional and Three-Dimensional Radiographs Using Clinically Relevant Parameters

**DOI:** 10.3390/dj7020050

**Published:** 2019-05-01

**Authors:** Julia C. Schmidt, Claudia-Julie Gutekunst, Dorothea Dagassan-Berndt, Patrick R. Schmidlin, Clemens Walter

**Affiliations:** 1 Department of Periodontology, Endodontology and Cariology, University Center for Dental Medicine, University of Basel, CH-4056 Basel, Switzerland; julia.schmidt@unibas.ch (J.C.S.); claudia-julie.gutekunst@unibas.ch (C.-J.G.); 2 Department of Oral Surgery, Oral Radiology and Oral Medicine, University Center for Dental Medicine, University of Basel, CH-4056 Basel, Switzerland; dorothea.dagassan@unibas.ch; 3 Clinic of Preventive Dentistry, Periodontology and Cariology, Center of Dental Medicine, University of Zurich, CH-8032 Zurich, Switzerland; patrick.schmidlin@zzm.uzh.ch

**Keywords:** diagnosis, radiographs, cone beam computed tomography, full-mouth intraoral radiograph, panoramic radiograph

## Abstract

This work compared the assessment of clinically relevant parameters by two-dimensional, that is, full-mouth intraoral radiograph (I-O) and panoramic radiograph (OPT), and three-dimensional, that is, cone beam computed tomography (CBCT), imaging methods. Different radiographic images (CBCT, I-O and OPT) were available for a 53-year-old female patient with dental and periodontal problems. A total of 14 dental and periodontal parameters were assessed by two independent examiners and compared among the three radiographic imaging modalities. For 10 parameters (71%), the CBCT images were superior to both I-O and OPT images. In contrast, CBCT demonstrated an inferior performance compared to I-O and OPT in the assessment of caries and dental restorations. Compared to OPT, I-O provided more clinically relevant findings for 10 out of 14 parameters (71%). Agreement between I-O and OPT was found with respect to dehiscence, fenestration, the number of bone walls and the root canal cross-section. Differences between the radiographic images were more likely to be detected when maxillary teeth rather than mandibular teeth were assessed with regard to furcation involvement, root proximity and root fusion.

## 1. Introduction

Conventional two-dimensional radiography is a standard diagnostic tool in dental medicine and may complement the medical history and clinical examination of a patient [1]. According to the particular indication, specific radiographic images are obtained, for example, panoramic radiograph (OPT), bitewing radiographs and/or periapical images of diseased teeth and periodontia. In patients with periodontitis, a full-mouth intraoral radiographic set (I-O) is prepared during the initial examination [2]. Radiographs are expected to support the diagnosis, prognosis, treatment planning, and/or the evaluation of treatment when applied.

During the last two decades, cone beam computed tomography (CBCT) has been introduced and increasingly used in different disciplines of dental medicine, including oral surgery, implantology, dental traumatology, orthodontics, endodontics and periodontology [3,4]. Three-dimensional CBCT may provide higher accuracy and a greater amount of information than two-dimensional imaging modalities. In the fields of endodontics and periodontology, CBCT has demonstrated advantages in the planning of apical surgery and in the detection of furcation involvement (FI) when invasive periodontal surgery is planned [5,6]. However, the use of CBCT is limited by its higher radiation doses and additional costs. Thus, CBCT should be restricted for use in cases when it would significantly benefit the patient [3,4].

The aim of the present case report was to compare the assessment of different clinically relevant parameters by two-dimensional, that is, I-O and OPT, and three-dimensional, that is, CBCT, radiographic imaging methods in a specific patient with periodontitis.

## 2. Materials and Methods

### 2.1. Patient Characteristics

**Figure 1:**
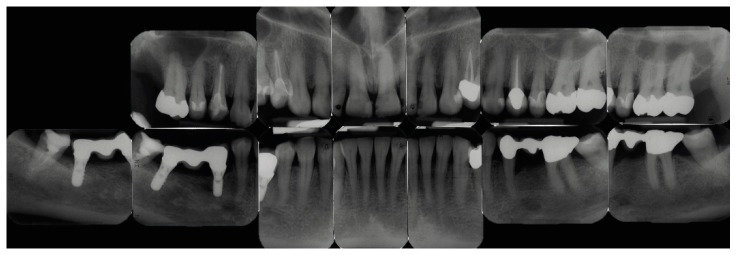
Full-mouth intra-oral radiographic set (I-O) represented by periapical films.

**Figure 2:**
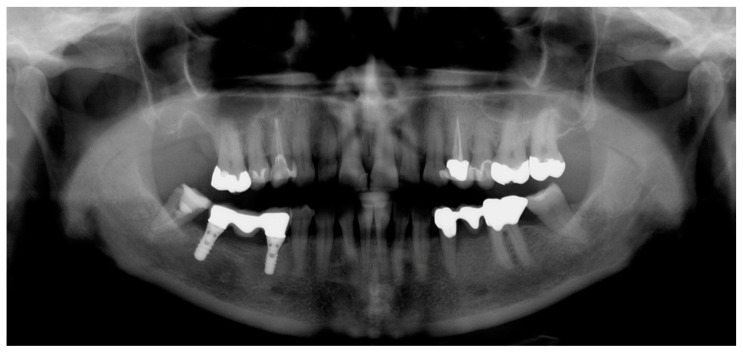
Panoramic radiograph (OPT).

**Figure 3:**
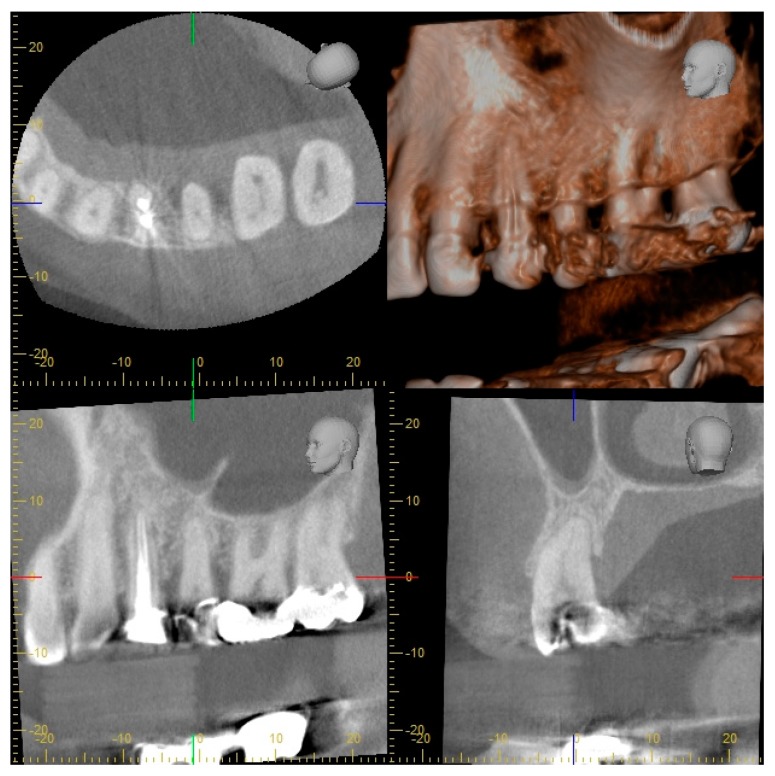
Part of full-mouth cone beam computed tomography (CBCT) with horizontal, sagittal and transversal sections of the first and second left maxillary premolars and molars.

A 53-year-old Caucasian woman presented to the Department of Periodontology, Endodontology and Cariology (University of Basel, Switzerland) for periodontal treatment. The patient gave her consent for publication of the patient material. The patient suffered from diabetes mellitus type 2 (HbA1c value, 7.1). Evaluation of her smoking history revealed that she was a smoker with a daily consumption of 10 cigarettes for 34 years, that is, approximately 17 pack years. Teeth 18, 17, 28, 35, 37 and 46 (FDI numbering system) were missing. In addition, teeth 45 and 47 had each been replaced by an implant. The clinical examination showed increased probing pocket depths (PPD) up to 7 mm, grade I FI and bleeding on probing of 23%. A misfit of the restorations on teeth 11 and 48 was detected and tooth 37 showed a cervical primary caries. All teeth positively responded to pulp sensitivity testing with CO_2_ with the exception of root canal treated teeth. An I-O was prepared for diagnostic reasons in our clinic (Figure 1). Approximately four weeks prior, OPT and full-mouth CBCT images had been conducted alio loco (Figure 2 and Figure 3). The periodontal diagnosis was severe localized chronic (smoker) periodontitis [7,8]. According to the current classification of periodontal diseases, the diagnosis was “stage III/grade B periodontitis” [9]. The caries diagnosis was primary caries on tooth 37.

Tooth 48 was extracted and teeth 11 and 37 were restored with composite fillings. Subsequently, nonsurgical periodontal treatment (systematic scaling and root planing) was performed on all affected teeth using ultrasonic devices and hand instruments under local anaesthesia. Revaluation of the periodontal conditions after three months demonstrated PPDs of < 6 mm. The patient was referred for supportive periodontal therapy (SPT) every three months. Follow-up examinations confirmed a stable situation (PPD 2–5 mm) almost eight years after initial periodontal treatment.

### 2.2. Radiographic Methods

I-O imaging consisted of 13 periapical films collected in parallel technique with I-O dental films (IP 22 Insight Doppel SP; Kodak GmbH, Stuttgart, Germany) using a film holder with 90° angulation (Rinn, Dentsply Rinn, Elgin, IL, USA) at 65 kV and 7 mA with an exposure time of 0.12–0.15 s (Dental EZ HDX, Dental EZ, Hertfordshire, UK) (Figure 1). Information about the technical parameters used for OPT and CBCT could be obtained retrospectively as follows: The OPT was conducted with a Cranex 3 system (Soredex, Orion Corporation, Finland) at 73 kV and 10 mA (Figure 2). CBCT was performed with a 3D Accuitomo 80 system (J. Morita, Kyoto, Japan) with a volume of 8 cm x 8 cm and at 90 kV and 5 mA with a voxel edge length of 0.160 mm (Figure 3).

### 2.3. Analysis of Radiographic Images

The radiographic (I-O, OPT and CBCT) images were analysed by two independent examiners (C.J.G. and J.C.S.). Each of the radiographic images was assessed in terms of 14 pre-defined dental and periodontal parameters (Table 1), which evaluated teeth, roots or implants, if applicable (e.g., FI in multirooted teeth) (Table 2). In accordance with validated and previously published classification and/or scoring systems, all assessed structures (teeth, roots, implants) received a respective score (Table 1 and Table 2). All teeth were assessed with the exception of tooth 48, as this tooth was not completely imaged by CBCT. The time between analysis of the three sets of radiographic images was at least one week for each. The examiners were calibrated by example radiographs before the start of the measurements. Teeth on which the examiners did not agree were discussed until concordance was reached.

According to established protocols, the analysis of the OPT and I-O images was performed using a light box, a dental loupe with a 2.5-fold magnification factor, a conventional loupe protected against lateral light incidence and a ruler with a millimetre scale [10,11]. The CBCT images were analysed in the axial, sagittal and coronal views using the 3D imaging software i-Dixel-3DX (J. Morita, Dietzenbach, Germany). Using this software, the image quality could be optimized and additional analyses were performed with different functions, such as angulation and length measurements. The size of the slides and the light and contrast could be also adjusted. Analysis of the CBCT images was performed using the same computer and monitor (58.5 cm, 1920 × 1080 pixels, OptiPlex 9030, Dell, Round Rock, USA) under standardized conditions in a darkened room. After parameter assessment, pairwise comparisons between the radiographic images were made (CBCT versus I-O, CBCT versus OPT, I-O versus OPT). The results of the comparisons were categorized as agreement (CBCT = I-O, CBCT = OPT, I-O = OPT), superiority (CBCT > I-O, CBCT > OPT, I-O > OPT) or inferiority (CBCT < I-O, CBCT < OPT, I-O < OPT) in the assessment of a specific parameter. For each parameter and comparison category, the absolute number of teeth was calculated (Table 3). For example, the findings of vertical bone defects on I-O and OPT imaging were comparable (I-O = OPT) in 22 teeth, while I-O showed more information (I-O > OPT) in 3 teeth. In the next step, the results of the comparisons for each parameter were summarized and the percentage distribution of each category was determined (Table 3). For example, I-O was superior to OPT in 12% of teeth in the assessment of vertical bone defects. CBCT was referred to as the gold standard when compared to the two-dimensional imaging modalities, for example, agreement was present if the findings were equal and CBCT superiority was present if the findings were different. Inferiority resulted if a parameter could not be evaluated by CBCT due to artefacts but could be evaluated by I-O and/or OPT. When comparing the two-dimensional imaging modalities, I-O served as the gold standard.

## 3. Results

The number of assessed structures per parameter—that is, teeth, implants, roots, root canals, furcations, vertical bone defects and restorations and the distribution of scores—are shown in Table 2. On CBCT, four out of 14 parameters (root canal anatomy, root canal cross-section, caries and restorations) were not assessable for any tooth or root. The assessment was primarily impaired due to artefacts, as well as resolution and/or contrast limitations. On I-O, the assessability of parameters was diminished in five out of 14 parameters, while four parameters (dehiscence, fenestration, number of bone walls and root canal cross-section) were not at all assessable. On OPT, the estimation was reduced in 12 out of 14 parameters. The impaired assessability of the two-dimensional radiographs was caused by overlapping effects, blurring and/or the missing third dimension.

The comparison of the three radiographic methods (CBCT, I-O and OPT) is shown in Table 3. CBCT was superior to I-O in 10 out of 14 parameters (71%), including dehiscence, fenestration, vertical bone defects including bone walls, FI, root proximity, root fusion, root canal anatomy, root canal cross-section and periapical status. Agreement between CBCT and I-O was found for two parameters (number of roots and root canal filling) and CBCT inferiority to I-O was found for two parameters (caries and restoration quality). Compared to OPT, CBCT was superior for 12 out of 14 parameters (86%) and inferior for two parameters (caries and restoration quality). The comparison between I-O and OPT showed I-O superiority for 10 out of 14 parameters (71%) and agreement for four parameters (29%), which were dehiscence, fenestration, the number of bone walls of vertical bone defects and root canal cross-section.

Differences between the radiographic images were more likely to be detected in maxillary teeth than mandibular teeth (Table 3). In contrast to the maxillary teeth, in the mandibular teeth, the agreement between CBCT and I-O (2 versus 4 parameters), CBCT and OPT (0 versus 4 parameters) and between I-O and OPT (4 versus 10 parameters) was higher.

Comparing all three radiographic methods together, CBCT superiority to both I-O and OPT was found for 10 out of 14 parameters (71%), including dehiscence, fenestration, vertical bone defects including bone walls, FI, root proximity, root fusion, root canal anatomy, root canal cross-section and periapical status. In contrast, CBCT demonstrated inferiority to I-O and OPT in the assessment of two parameters (14%) (caries and restoration quality).

## 4. Discussion

In the present case report, the assessability of dental and periodontal parameters by three different radiographic methods was exemplarily compared. While CBCT was superior to both two-dimensional radiographic methods (10 out of 14 parameters), I-O showed superiority to OPT (10 out of 14 parameters). Differences between the radiographic methods were more likely to be detected when maxillary teeth than mandibular teeth were assessed. This finding might be explained by the root anatomy of maxillary teeth, that is, the presence of a palatal root, which is difficult or even impossible to assess on two-dimensional radiographs. In addition, the diagnostic accuracy of the radiographic methods differed if used for diagnosing caries or assessing endodontic or periodontal parameters.

Diagnostic accuracy needs to be discussed, when digital or film-based radiographs were considered. Film-based I-O (E/F-speed film) show a similar spatial resolution to digital systems. However, further developments in digital technologies focus on dose reduction and the dynamic range of images, in particular adjustment of brightness and contrast, in order to enhance the image and the visibility of relevant structures, relevant for diagnosis. For panoramic radiographs, a recent study could show that film-based OPT could detect pathologies in a similar way as obtained by digital OPT [23]. In addition, the ALARA-principle should be taken into account, where accessible radiographs are used, instead of acquiring new radiographs [24]. For three-dimensional radiological diagnoses, the parameters for CBCT scan need to be adopted in relation to the clinical questions applied. This includes the size of anatomical structures. Adequate parameter adjustment can be acquired with different CBCT-machines as long as different fields of view and different voxel sizes are available [25].

Regarding caries diagnosis and restoration assessability, I-O showed the highest accuracy (I-O > OPT > CBCT). The diagnostic value of CBCT was limited by the occurrence of beam-hardening artefacts and streaks from restorations and implants. Some previous studies have investigated the application of CBCT for caries diagnosis [26,27,28,29]. All in all, there was no evidence showing benefits of CBCT compared to I-O modalities in evaluating the coronal tooth structure and diagnosing caries. However, some authors found equivalent diagnostic accuracy in caries detection [30,31,32,33,34]. This outcome might be influenced by ex vivo study designs with extracted human teeth not or minimally restored and/or artificial caries lesions extending to the dentin. Therefore, artefacts might be kept to a minimum and advanced caries lesions might easily be detected. In contrast, the present case report reflects a true clinical situation with various fillings, prosthetic restorations and implants. In accordance with previous studies, the diagnostic value of OPT in caries detection was inferior to that of I-O [35,36,37]. Thus, according to actual guidelines, I-O radiographic examination remains the gold standard for detecting carious lesions in daily practice [4].

With respect to the detectability of root canal morphology and periapical lesions, CBCT provided the most information, while I-O demonstrated advantages over OPT (CBCT > I-O > OPT). This finding is in line with those of previous studies assessing root anatomy and canal morphology by CBCT [38,39,40,41]. It has been shown that additional roots and/or complex root canal structures, for example, C-shaped canals or second mesiobuccal canals in maxillary molars, can be detected using high-resolution CBCT. In the present case, second mesiobuccal canals were identified in maxillary first molars. However, the resolution capacity may limit the detection of narrow accessory and/or obliterated root canals, root fractures and root perforations [42]. In addition, the evaluation of root canal morphology and/or root canal fillings may be impaired by artefacts from restorations, root canal posts and other filling materials. In the present case, CBCT showed slightly more roots with periapical pathosis than did the two-dimensional imaging modalities. Similar findings were reported in previous studies, that is, more apical lesions were detected by CBCT than by periapical images during preoperative diagnostic [43,44,45,46,47,48] and postoperative examinations [49]. However, a final diagnosis can be established by either two-dimensional or three-dimensional images, as the differentiation of granulomas and radicular cysts is not possible [50]. Thus, the radiographic finding of a periapical lesion should be regarded in the context of the clinical findings to estimate the relevance of any apical radiolucency. In the present case, no additional endodontic treatment was performed as clinical signs of pathosis were lacking.

Dehiscences and fenestrations could not be identified on two-dimensional (I-O and OPT) radiographs. In contrast, assessment of the buccal alveolar bone was possible by three-dimensional CBCT. While the assessment of vertical bone defects seemed to be more reliable by CBCT and I-O (CBCT > I-O > OPT) in the present case report, the number of bone walls could be evaluated exclusively by the three-dimensional method. Vertical bone defects constitute a risk factor for further attachment loss and are relevant for the assessment of the tooth-related prognosis [13]. In two case series with a total of 40 patients, the assessability of vertical bone defects was compared between CBCT and periapical images [51,52]. These studies showed significant differences in the alveolar bone height between measurements based on the CBCT and periapical images in favour of the three-dimensional method. In contrast, no differences were found regarding the depth or size of the bone defects. The morphology and the number of bone walls may influence the use of materials in regenerative surgery. However, until now, there has been no evidence for the use of CBCT in the diagnosis and treatment planning of vertical bone defects [6,53]. Thus, the standard use of CBCT for the assessment of vertical bone defects is not recommended [4]. In this patient, a CBCT was available. However, periapical radiographic imaging was performed additionally due to current guidelines in periodontology and the possibility of comparing future follow-up radiographs [4,53]. The herein observed superiority of I-O to OPT may confirm the benefit of periapical images for the prognosis and treatment planning of vertical bone defects [2]. In addition, CBCT and I-O showed superiority in the evaluation of horizontal, that is, furcation, defects, root proximity and root fusion in maxillary teeth (CBCT > I-O > OPT). The risk of progressive attachment loss and consequent tooth loss is increased in teeth with FI [54]. Thus, multirooted teeth with FI have a worse long-term prognosis than single-rooted teeth or teeth without FI. During nonsurgical periodontal treatment, the radiological furcation diagnosis is usually made by I-O [1,4]. After nonsurgical treatment, however, teeth with FI may show an increased PPD and thus the need for surgery [55,56]. The planning of surgical therapy requires the adequate diagnostic evaluation of the interfurcal bone loss, inter- and periradicular bone around every root and root morphology [57]. The assessability of and/or overlapping effects on two-dimensional radiographs impair the analysis of FI. In contrast, CBCT enables adequate assessment of the maxillary periodontal situation for treatment planning [6,11,58]. The indication for CBCT of the maxillary molars as an additional diagnostic tool depends on the clinical invasiveness of the planned therapy, that is, a cost-intensive root canal treatment prior to resective surgery could be justification for CBCT [6,59].

A strength of the present case report was the availability of three different radiographic images for comparison. This setup with three methods seems to be, for ethical and practical reasons, not feasible in a controlled clinical study. In addition, the patient provided real clinical conditions with a combination of dental and periodontal problems by which clinicians are usually confronted in daily practice.

Several dental and periodontal parameters were assessed herein. In general, it is mandatory to meticulously examine the entire radiographic image which is produced to search for all relevant findings including dental and non-dental structures. With the introduction of CBCT, the reading of images has become more extensive and a skilled experience is required for the interpretation and diagnosis of the three-dimensional images [3].

## 5. Conclusions

In conclusion, the present case report demonstrates that CBCT findings add substantial information regarding different periodontal and endodontic parameters, while two-dimensional images are beneficial regarding caries diagnosis. Nevertheless, the application of CBCT should remain limited to difficult and complex cases in which diagnosis and decision making are challenging by conventional diagnostics. Any use of radiographic imaging should be critically and individually evaluated with regard to the particular problem and in view of the radiation exposure, additional costs and benefit-risk ratio.

## Figures and Tables

**Table 1:** Dental and periodontal parameters, related scoring system/classification and criteria.

Parameter	Scoring System/Classification		Criteria for Scoring/Classification of Parameters	
Number of roots	1	1 root	• complete root fusion = 1 root • incomplete root fusion = multirooted teeth (≥2 roots)	
	2	2 roots		
	3	3 roots		
Dehiscence	0	not present	• distance between alveolar crest and CEJ > 2 mm, resulting in exposed cervical root surface [12], buccally assessed	
	1	present		
Fenestration	0	not present	• lack of alveolar bone, not affecting the marginal alveolar bone and resulting in exposed root surface [12], buccally assessed	
	1	present		
Vertical bone defect	0	not present	• apical location of the base of the pocket with regard to the residual alveolar crest [13,14] • intrabony = infrabony component of the defect affecting one tooth/implant • crater = defect affecting two adjacent root/implant surfaces	
	1	intrabony defect		
	2	crater		
Number of bone walls	1	one-wall defect	• number of residual alveolar bone walls in intrabony defects [14]	
	2	two-wall defect		
	3	three-wall defect		
	4	combination defect		
Furcation involvement	0	not present	• horizontal loss of periodontal tissue support, that is, radiolucency in the furcation area [15] • classification according to Hamp et al. [15]	
	1	present (I, II, III)	I	horizontal loss of periodontal tissue support ≤ 3 mm
			II	horizontal loss > 3 mm but not “through and through” destruction
			III	horizontal “through and through” tissue destruction in the furcation
Root fusion	0	not present	• lack of a separating periodontal ligament between two adjacent roots [11]	
	1	present		
Root proximity	0	not present	• separating periodontal ligaments between two adjacent roots ≤ 0.8 mm [16]	
	1	present		
Root canal anatomy	I	1 canal, 1 foramen	• classification according to Vertucci [17] • evaluation of each root separately	
	II	2 canals, fusion, 1 foramen		
	III	1 canal, division, fusion, 1 foramen		
	IV	2 canals, no fusion, 2 foramina		
	V	1 canal, division, 2 foramina		
	VI	2 canals, fusion, division, 2 foramina		
	VII	1 canal, division, fusion, division, 2 foramina		
	VIII	3 canals, no fusion, 3 foramina		
Root canal cross-section	1	round	• classification according to Jou et al. [18] • evaluation of each root canal separately	
	2	oval		
	3	long oval		
	4	flattened		
	5	irregular		
Periapical status	1	healthy (PAI 1, 2)	• scoring system according to Ørstavik et al. [19]	
			PAI 1	normal periapical structure
			PAI 2	small changes in bone structure
			PAI 3	changes in bone structure with mineral loss
	2	diseased (PAI 3, 4, 5)	PAI 4	apical periodontitis with well-defined radiolucent areas
			PAI 5	severe apical periodontitis with exacerbating features
			• multirooted teeth were given the highest score detected at any root [10]	
Root canal filling	0	insufficient (score >1)	• scoring system according to Weiger et al. [20] • length: (1) 0-2 mm short of the radiographic apex, (2) >2 mm short of the radiographic apex, (3) extruded beyond the radiographic apex • density: (1) no voids and close adaptation to root canal walls, (2) voids or insufficient adaptation	
	1	sufficient (score =1)		
Caries	R0	sound (no radiolucency)	• scoring system according to Pitts [21] • evaluation of each tooth surface separately • each tooth was given the highest score detected at any tooth surface	
	R1	outer half enamel lesion		
	R2	inner half enamel lesion		
	R3	outer half dentin lesion		
	R4	inner half dentin lesion		
Restoration quality	0	intact	• scoring system according to Tronstad et al. [22] • not intact = sign of overhangs, recurrent decay or open margins • intact = any restoration that appeared intact radiographically	
	1	not intact		
In addition to all scores:	N not assessable		• for example, due to artefacts, overlapping effects, resolution capacity and/or contrast limitations	

CEJ, cemento-enamel junction; PAI, periapical index.

**Table 2:** Number of assessed structures and distribution of the scores of dental and periodontal parameters.

Parameter	Number of Assessed Structures	Distribution of the Scores of Parameters (Number of Structures) *		
		CBCT	I-O	OPT
Number of roots	23 teeth	1 (19), 2 (1), 3 (3)	1 (19), 2 (1), 3 (3)	1 (19), 2 (1), 3 (1), N (2)
Dehiscence	27 roots	0 (16), 1 (11)	N (27)	N (27)
Fenestration	27 roots	0 (18), 1 (9)	N (27)	N (27)
Vertical bone defect	23 teeth, 2 implants	0 (22), 1 (3)	0 (20), 1 (3), 2 (2)	0 (23), 2 (2)
Number of bone walls	2-5 vertical defects	3 (1), 4 (2)	N (5)	N (2)
Furcation involvement	11 furcation entrances	0 (11)	0 (4), N (7)	0 (1), N (10)
Root fusion	10 pairs of roots	0 (10)	0 (4), N (6)	0 (2), N (8)
Root proximity	10 pairs of roots	0 (8), 1 (2)	0 (2), 1 (2), N (6)	0 (1), 1 (1), N (8)
Root canal anatomy	30 roots	I (24), II (4), N (2)	I (18), II (2), N (10)	I (16), II (2), N (12)
Root canal cross-section	34 root canals	1 (30), N (4)	N (34)	N (34)
Periapical status	30 roots, 2 implants	1 (30), 2 (2)	1 (31), 2 (1)	1 (21), N (11)
Root canal filling	2 root canal fillings	1 (2)	1 (2)	0 (1), 1 (1)
Caries	23 teeth	R0 (6), R3 (1), N (16)	R0 (23)	R0 (16), N (7)
Restoration quality	16 restorations	0 (2), N (14)	0 (14), 1 (1), N (1)	0 (4), 1 (1), N (11)

*, see Table 1 for definition of scores.

**Table 3:** Comparison of the results of parameters between the radiographic methods (CBCT versus I-O, CBCT versus OPT, I-O versus OPT), classified as agreement (CBCT = I-O, CBCT = OPT, I-O = OPT), superiority (CBCT > I-O, CBCT > OPT, I-O > OPT) or inferiority (CBCT < I-O, CBCT < OPT, I-O < OPT). For each parameter, the number of all structures (e.g., teeth, roots) assessed, the number of maxillary and mandibular structures separately (in parentheses) and the summary of pairwise (percentage portion of the determining category; highlighted in light grey) and overall comparisons (CBCT versus I-O versus OPT; highlighted in dark grey) are shown.

	CBCT versus I-O				CBCT versus OPT				I-O versus OPT				CBCT vs. I-O vs. OPT
	CBCT = I-O	CBCT > I-O	CBCT < I-O	Overall	CBCT = OPT	CBCT > OPT	CBCT < OPT	Overall	I-O = OPT	I-O > OPT	I-O < OPT	Overall	Overall
Number of roots ^1^	23 (13, 10)	0	0	CBCT = I-O (100)	21 (11, 10)	2 (2, 0)	0	CBCT > OPT (9)	21 (11, 10)	2 (2, 0)	0	I-O > OPT (9)	CBCT = I-O > OPT
Dehiscence	0	27 (16, 11)	0	CBCT > I-O (100)	0	27 (16, 11)	0	CBCT > OPT (100)	27 (16, 11)	0	0	I-O = OPT (100)	CBCT > I-O = OPT
Fenestration	0	27 (16, 11)	0	CBCT > I-O (100)	0	27 (16, 11)	0	CBCT > OPT (100)	27 (16, 11)	0	0	I-O = OPT (100)	CBCT > I-O = OPT
Vertical bone defect ^2^	22 (11, 11)	3 (2, 1)	0	CBCT > I-O (12)	21 (10, 11)	4 (3, 1)	0	CBCT > OPT (16)	22 (12, 10)	3 (1, 2)	0	I-O > OPT (12)	CBCT > I-O > OPT
Number of bone walls	0	3 (2, 1)	0	CBCT > I-O (100)	0	1 (1, 0)	0	CBCT > OPT (100)	2 (2, 0)	0	0	I-O = OPT (100)	CBCT > I-O = OPT
Furcation involvement ^3^	4 (3, 1)	7 (6, 1)	0	CBCT > I-O (64)	1 (0, 1)	10 (9, 1)	0	CBCT > OPT (91)	8 (6, 2)	3 (3, 0)	0	I-O > OPT (27)	CBCT > I-O > OPT
Root fusion	4 (3, 1)	6 (6, 0)	0	CBCT > I-O (60)	2 (1, 1)	8 (8, 0)	0	CBCT > OPT (80)	8 (7, 1)	2 (2, 0)	0	I-O > OPT (20)	CBCT > I-O > OPT
Root proximity	4 (3, 1)	6 (6, 0)	0	CBCT > I-O (60)	2 (1, 1)	8 (8, 0)	0	CBCT > OPT (80)	8 (7, 1)	2 (2, 0)	0	I-O > OPT (20)	CBCT > I-O > OPT
Root canal anatomy	21 (12, 9)	9 (7, 2)	0	CBCT > I-O (30)	19 (10, 9)	11 (9, 2)	0	CBCT > OPT (37)	28 (17, 11)	2 (2, 0)	0	I-O > OPT (7)	CBCT > I-O > OPT
Root canal cross-section	5 (4, 1)	29 (18, 11)	0	CBCT > I-O (85)	5 (4, 1)	29 (18, 11)	0	CBCT > OPT (85)	34 (22, 12)	0	0	I-O = OPT (100)	CBCT > I-O = OPT
Periapical status	31 (18, 13)	1 (1, 0)	0	CBCT > I-O (3)	19 (6, 13)	13 (13, 0)	0	CBCT > OPT (41)	20 (7, 13)	12 (12, 0)	0	I-O > OPT (38)	CBCT > I-O > OPT
Root canal filling	2 (2, 0)	0	0	CBCT = I-O (100)	1 (1, 0)	1 (1, 0)	0	CBCT > OPT (50)	1 (1, 0)	1 (1, 0)	0	I-O > OPT (50)	CBCT = I-O > OPT
Caries	6 (1, 5)	1 (0, 1)	16 (12, 4)	CBCT < I-O (70)	11 (6, 5)	2 (1, 1)	10 (6, 4)	CBCT < OPT (43)	16 (6, 10)	7 (7, 0)	0	I-O > OPT (30)	I-O > OPT > CBCT
Restoration quality	2 (1, 1)	1 (1, 0)	13 (9, 4)	CBCT < I-O (81)	12 (9, 3)	1 (1, 0)	3 (1, 2)	CBCT < OPT (19)	6 (3, 3)	10 (8, 2)	0	I-O > OPT (63)	I-O > OPT > CBCT

^1^ Example (CBCT versus I-O): in 23 teeth (13 maxillary teeth and 10 mandibular teeth), the same number of roots was assessable by CBCT and I-O (agreement, that is, CBCT = I-O). In no teeth was CBCT either superior (CBCT > I-O) or inferior (CBCT < I-O) regarding the number of roots compared to I-O. Thus, agreement between CBCT and I-O was present in 100% of assessed teeth. ^2^ Example (I-O versus OPT): in 22 teeth (12 maxillary teeth and 10 mandibular teeth), the assessability of the bone regarding vertical defects was equal for I-O and OPT (agreement, that is, I-O = OPT). In 3 teeth (one maxillary tooth and two mandibular teeth), I-O showed superiority to OPT (I-O > OPT) and in no teeth, I-O showed inferiority (I-O < OPT) to OPT. Thus, superiority of I-O to OPT was present in 12% of assessed teeth. ^3^ Example (CBCT versus I-O versus OPT): CBCT showed superiority to I-O and OPT, while I-O provided more information than OPT (CBCT > I-O > OPT).
